# Re-resection at first recurrence is associated with prolonged survival in young patients with high-grade glioma: A multi-center study

**DOI:** 10.1093/noajnl/vdag237

**Published:** 2026-09-07

**Authors:** David A Giles, Jing Wang, Markus Anzaldua-Campos, Uma Mani, Ryan Harrigan, Kaethe Leonard, Bethany Y Norton, Breanne Reisen, Diana Chang, Pavan Guttipatti, Sage Rahm, Anastasia Arynchyna-Smith, James M Johnston, Todd Hankinson, Anthony C Wang, Michael Dewan, Peter Chiarelli, Jason Hauptman, Jennifer M Strahle, Jarod L Roland, Michael DeCuypere, Eric M Thompson

**Affiliations:** Taylor Family Department of Neurosurgery, WashU Medicine, St. Louis, Missouri, USA; The Brain Tumor Center at Siteman Cancer Center, St. Louis, Missouri, USA; Taylor Family Department of Neurosurgery, WashU Medicine, St. Louis, Missouri, USA; Taylor Family Department of Neurosurgery, WashU Medicine, St. Louis, Missouri, USA; Taylor Family Department of Neurosurgery, WashU Medicine, St. Louis, Missouri, USA; Taylor Family Department of Neurosurgery, WashU Medicine, St. Louis, Missouri, USA; Ann & Robert H. Lurie Children’s Hospital of Chicago, Chicago, Illinois, USA; Phoenix Children’s Hospital, Phoenix, Arizona, USA; Monroe Carell Jr. Children’s Hospital at Vanderbilt, Nashville, Tennessee, USA; Department of Neurosurgery, University of California Los Angeles, Los Angeles, California, USA; Children’s Hospital Colorado, Aurora, Colorado, USA; Department of Neurosurgery, University of Alabama, Birmingham, Alabama, USA; Department of Neurosurgery, University of Alabama, Birmingham, Alabama, USA; Department of Neurosurgery, University of Alabama, Birmingham, Alabama, USA; Children’s Hospital Colorado, Aurora, Colorado, USA; Department of Neurosurgery, University of California Los Angeles, Los Angeles, California, USA; Ann & Robert H. Lurie Children’s Hospital of Chicago, Chicago, Illinois, USA; Phoenix Children’s Hospital, Phoenix, Arizona, USA; Phoenix Children’s Hospital, Phoenix, Arizona, USA; Taylor Family Department of Neurosurgery, WashU Medicine, St. Louis, Missouri, USA; The Brain Tumor Center at Siteman Cancer Center, St. Louis, Missouri, USA; Taylor Family Department of Neurosurgery, WashU Medicine, St. Louis, Missouri, USA; Monroe Carell Jr. Children’s Hospital at Vanderbilt, Nashville, Tennessee, USA; Taylor Family Department of Neurosurgery, WashU Medicine, St. Louis, Missouri, USA; The Brain Tumor Center at Siteman Cancer Center, St. Louis, Missouri, USA

**Keywords:** children, high-grade glioma, pediatric brain tumor, recurrence, resection

## Abstract

**Background:**

Surgical resection remains standard of care for high-grade glioma. It is unclear, however, whether re-resection at the time of first recurrence confers further survival benefit in young patients.

**Methods:**

This multi-center, retrospective cohort study included patients age 0-21 years with supratentorial high-grade glioma who underwent gross total or near total resection at time of initial diagnosis which subsequently recurred. Patients were grouped by therapeutic re-resection at first recurrence. Post-recurrence overall survival was assessed with Kaplan-Meier methods, multivariable Cox regression, and propensity score-matched analyses.

**Results:**

Forty-four patients with a median age of 12.5 years from 7 institutions were included. 61% (*n* = 27) underwent re-resection and 39% (*n* = 17) did not. The 2 groups (re-resection and no re-resection) did not significantly differ in sex, age at recurrence, functional status, pathology, chemotherapy and radiation exposure, or institution (all *P* > .05). Kaplan-Meier analysis demonstrated significantly longer post-recurrence overall survival with re-resection compared with no re-resection (median 24.6 vs 8.5 months; log-rank *P* = .0001). Patients who underwent re-resection had a 73% lower hazard of death after recurrence compared with patients who did not undergo re-resection by multivariable Cox regression (HR 0.27, 95% CI, 0.10-0.69; *P* = .006) or by propensity score matching analyses (HR 0.27, 95% CI, 0.11-0.68; *P* = .005).

**Conclusions:**

Therapeutic re-resection at first recurrence was associated with improved post-recurrence survival, which remained significant after multivariable adjustment or propensity score matching. This study provides evidence for re-resection in recurrent high-grade glioma in young patients.

Key Points

- Re-resection at recurrence is associated with prolonged survival in young patients with HGG.
- The survival association is most pronounced in WHO Grade 3 gliomas.
- The survival association remains significant after propensity score matching.

Importance of the Study

Maximal safe resection is the standard of care for high-grade glioma. Limited data has been available to guide whether to proceed with repeat resection at the time of recurrence in young patients. This is the first multi-institutional comprehensive analysis of re-resection in patients age 0-21 years with supratentorial high-grade glioma, and it reveals a significant survival benefit associated with re-resection at first recurrence.

Brain cancer is the most common cause of cancer death in patients age 0-14^1^ and improvements in survival remain elusive. High-grade glioma (HGG, World Health Organization Grade 3 or 4) is particularly devastating in young patients, with an approximate median progression-free survival (PFS) of 10 months and overall survival (OS) of 17 months.^2^ Standard treatment includes maximal safe resection followed by radiation +/− chemotherapy.^3^ Several studies have shown a survival benefit associated with increased extent of initial resection, particularly in children who have had a gross total resection (GTR) for malignant brain tumors at the time of initial diagnosis^2^^,^^4^^,^^5^; however, it is unknown if re-resection at first recurrence impacts OS.

Our study focuses on the clinical scenario where near total or GTR was performed at diagnosis, but a local recurrence is noted at a later date. Few treatment options are available at the time of recurrence, and survival is very poor.^6^ There is evidence to support re-resection at progression/recurrence in adult low-grade^7^ and HGG^8^; however, there is limited data to guide surgical decision-making in younger patients. This multi-center retrospective cohort study addresses the critical clinical question of whether re-resection confers a survival benefit in young patients with HGG.

## Methods

The study was approved by the Washington University School of Medicine Institutional Review Board (IRB#202404150) as well as the review boards of each participating institution. As a retrospective study, the requirement for informed consent was waived by the IRB. Clinical data was collected at each individual institution and sent to Washington University School of Medicine for centralized analysis.

### Study Population

This is a retrospective study of patients with HGG initially diagnosed between 2000 and 2023 and age 0 to 21 years at time of diagnosis. HGG was defined as WHO Grade 3 or 4 as well as those identified as “high grade” on the pathology report if WHO Grade was not otherwise specified (nos). As such, it is inclusive of all HGG, not only those defined as pediatric-type HGGs in WHO 2021.^9^ To refine to only those for which maximal safe surgical resection was feasible, only tumors in the frontal, temporal, parietal, or occipital lobes (not brainstem, cerebellum, basal ganglia, or thalamus) were included. All patients underwent initial resection at diagnosis with either a gross total resection (GTR: no residual T1 + C or FLAIR lesion) or near total resection (NTR: ≥90% resected of T1 + C or FLAIR lesion) based on postoperative MRI. If initial surgery was a biopsy, definitive initial resection must have been completed within 2 months of diagnosis. If multiple resections were required to achieve GTR/NTR, these must also have been completed within 2 months of diagnosis and were not included as re-resections. Patients must have a documented recurrence and a minimum of 2 months follow-up after diagnosis of recurrence. Therapeutic re-resection was defined as intent for maximum safe resection. Re-resection extent of resection was defined as subtotal resection (STR: <90% resected of T1 + C or FLAIR lesion), NTR, and GTR.

### Clinical Data Collection

Clinical characteristics (date of diagnosis and recurrence, sex, age, functional status), tumor characteristics (location, WHO Grade, molecular characteristics), and treatment characteristics (number of radiation or chemotherapy regimens) were obtained from chart review. Date of initial diagnosis or recurrence were determined based on clinical notes or radiography report. Functional status was assessed based on clinical notes using the Karnofsky performance status for patients 16 years of age or older^10^ or Lansky performance status for those under 16 years of age.^11^ For simplicity, the abbreviation KL is used to refer to either functional status throughout the text. Given high variability in protocols, adjuvant chemotherapy and radiotherapy treatments were simply quantified as the aggregate number of treatment regimens based on clinical notes. For BRAF status, patients were considered positive if a BRAF mutation was reported in molecular information or if the patient received a BRAF or MEK inhibitor (dabrafenib, tovorafenib, vemurafenib, binimetinib, tramatenib, cobimetinib). An anonymized subset of the data has been compiled in Supplementary Table S1.

### Statistics

Descriptive statistics were generated to compare groups by re-resection status. Continuous variables were summarized as mean ± standard deviation (SD) and median with interquartile range (IQR) and compared using Student’s t test or Wilcoxon rank-sum test, as appropriate. Categorical variables were presented as frequencies and percentages and compared using Chi-square or Fisher’s exact tests. A multivariable Cox proportional hazards model was fit with re-resection group as the main predictor, adjusting for age at recurrence (categorized as ≤13 vs >13), sex, WHO Grade category, post-recurrence chemotherapy regimen count, post-recurrence radiation regimen count (categorized as 0 vs ≥1), and Karnofsky/Lansky (KL) score at first recurrence. Proportional hazards assumptions were assessed using the supremum test. Hazard ratios (HR) with 95% CIs and *P*-values were reported. To further address confounding by indication, we estimated each patient’s propensity for re-resection using logistic regression including the same covariates. We performed 1:1 greedy nearest-neighbor matching on the logit of the propensity score and assessed covariate balance using standardized mean differences. In the propensity score-matched cohort, OS after recurrence was compared between groups using Kaplan-Meier curves with log-rank testing and Cox regression with robust sandwich standard errors clustered by matched pair. Analyses were conducted in SAS 9.4; two-sided *P* < .05 denoted statistical significance.

## Results

### Patient Characteristics

Among 44 patients with recurrent HGG, 61.4% (*n* = 27) underwent therapeutic re-resection and 38.6% (*n* = 17) had no re-resection. A list of the number of patients contributed by each institution can be found in Supplementary Table S2. Patients were distributed across multiple sites with no statistically significant difference between groups (*P* = .545). The 2 groups were broadly comparable on measured baseline characteristics (Table 1). Sex distribution was similar (no re-resection: 64.7% male vs re-resection: 55.6% male; *P *= 0.75). Age at recurrence did not differ (no re-resection: 13.5 ± 5.0 years; median 14.2 [IQR 12.6-16.0] vs re-resection: 13.1 ± 5.2 years; median 12.5 [IQR 10.3-17.1]; *P *= .40). There were more Grade 4 gliomas in the no re-resection group, but this did not reach statistical significance (no re-resection: 23.5% Grade 3, 76.5% Grade 4, 0% HGG nos vs re-resection: 40.7% Grade 3, 51.9% Grade 4, 7.4% HGG nos; *P *= .27). A similar percentage of patients also required a “second look” surgery to achieve NTR/GTR at the time of initial resection (17.6% no re-resection, 14.8% re-resection).

**Table 1.** Baseline clinical and demographic characteristics by re-resection group (re-resection vs. no re-resection)

Characteristic	Re-res (*n* = 27)	No Re-res (*n* = 17)	*P*-value
Sex			.75
Male	15 (55.6%)	11 (64.7%)	
Female	12 (44.4%)	6 (35.3%)	
Age at recurrence (years) (mean ± SD; Median [IQR])	13.1 ± 5.2; 12.5 [10.3-17.1]	13.5 ± 5.0; 14.2 [12.6-16.0]	.57
KL score at presentation (mean ± SD; Median [IQR])	90.0 ± 8.3; 90 [80-100]	89.4 ± 13.0; 90 [80-100]	.76
KL score at first recurrence (mean ± SD; Median [IQR])	89.6 ± 10.5; 90 [90-100]	87.1 ± 11.6; 90 [80-90]	.45
Pathology			.27
Grade 3	11 (40.7%)	4 (23.5%)	
Grade 4	14 (51.9%)	13 (76.5%)	
High-Grade NOS	2 (7.4%)	0 (0.0%)	
Chemotherapy regimen count			
Total (mean ± SD; Median [IQR])	3.8 ± 2.8; 3 [1-5]	3.7 ± 1.2; 4 [3-4]	.61
Prior to recurrence (mean ± SD; Median [IQR])	1.1 ± 0.9; 1 [0-2]	1.5 ± 0.9; 1.5 [1-2]	.19
Following recurrence (mean ± SD; Median [IQR])	2.7 ± 2.3; 2 [1-4]	2.2 ± 1.1; 2 [1-3]	.91
Radiotherapy regimen count			
Total (mean ± SD; Median [IQR])	1.6 ± 0.8; 2 [1-2]	1.6 ± 0.8; 2 [1-2]	.82
Prior to recurrence (mean ± SD; Median [IQR])	0.8 ± 0.5; 1 [0-1]	1.1 ± 0.4; 1 [1-1]	.07
Following recurrence (mean ± SD; Median [IQR])	0.9 ± 0.7; 1 [0-1]	0.5 ± 0.7; 0 [0-1]	.10
Time from diagnosis to first recurrence (months) (mean ± SD; Median [IQR])	21.1 ± 23.6; 14.4 [4.0-24.0]	12.3 ± 7.9; 10.0 [7.2-13.3]	.58
Time from definitive resection to first recurrence (months) (mean ± SD; Median [IQR])	20.6 ± 23.6; 14.2 [3.6-21.5]	12.1 ± 8.0; 9.7 [7.0-13.3]	.60
Survival status at last follow-up			.20
Dead	17 (63.0%)	14 (82.4%)	
Alive/censored	10 (37.0%)	3 (17.6%)	

Abbreviations: SD = standard deviation; IQR = interquartile range (Q1-Q3); KL = Karnofsky/Lansky performance score; NOS = not otherwise specified.

Categorical *P*-values from Fisher’s exact tests; continuous *P*-values from Wilcoxon rank-sum tests (as available).

The time from diagnosis to first recurrence did not differ significantly between groups (re-resection: 21.1 ± 23.6 months; median 14.4 [IQR 4.0-24.0] vs no re-resection: 12.3 ± 7.9 months; median 10.3 [IQR 7.2-13.3]; *P* = .58). The time from definitive resection to first recurrence also did not significantly differ between groups (re-resection: 20.6 ± 23.6 months; median 14.2 [IQR 3.6-21.5] vs no re-resection: 12.1 ± 8.0 months; median 9.7 [IQR 7.0-13.3]; *P* = .60).

Approximately 75% of patients had reported molecular information (Supplementary Tables S1 and S3). The mutational profiles were similar across groups with the exception of BRAF which was enriched in the re-resection group. In order to more accurately capture BRAF status given incomplete molecular information, patients who received a BRAF or MEK inhibitor were also identified (re-resection: 8 treated with inhibitors, 5 reported mutations; no re-resection: 3 treated with inhibitors, 1 reported mutation). All patients with reported BRAF mutations were also treated with BRAF/MEK inhibitors. BRAF status was thus defined as treatment with a BRAF/MEK inhibitor and was included in survival and multivariate analysis.

Functional status as measured by KL score was high and similar between groups. At presentation, mean KL scores were 90.0 ± 8.3 in the re-resection group and 89.4 ± 13.0 in the no re-resection group (median 90 [IQR 80-100] in both groups; *P* = .76). At first recurrence, KL scores remained comparable (re-resection: 89.6 ± 10.6; median 90 [IQR 80-100] vs no re-resection: 87.1 ± 11.6; median 90 [IQR 80-90]; *P* = .45), indicating similar functional performance at the time of recurrence and supporting comparability between groups with respect to baseline performance status.

Chemotherapy and radiotherapy exposure relative to resection were similar between groups. The total number of chemotherapy regimens did not differ between the re-resection and no re-resection groups (re-resection: 3.8 ± 2.8; median 3 [IQR 1-5] vs no-re-resection: 3.7 ± 1.2; median 4 [IQR 3-4]; *P* = .61). There was also no difference when considering chemotherapy regimen counts prior to recurrence (re-resection: 1.1 ± 0.9; median 1 [IQR 0-2] vs no re-resection: 1.5 ± 0.9; median 1.5 [IQR 1-2]; *P* = .19) or following recurrence (re-resection: 2.7 ± 2.3; median 2 [IQR 1-4] vs no re-resection: 2.2 ± 1.1; median 2 [IQR 1-3]; *P* = .91). Similarly, the total number of radiotherapy regimens was similar between the re-resection and no re-resection groups (both mean 1.6 ± 0.8; median 2 [IQR 1-2]; *P* = .82). There were more radiation naïve patients in the re-resection group compared to no re-resection (re-resection 7/27 vs no re-resection 1/17); however, there was no significant difference when considering radiotherapy regimen counts prior to recurrence (re-resection: 0.8 ± 0.5; median 1 [IQR 0-1] vs no re-resection: 1.1 ± 0.4; median 1 [IQR 1-1]; *P* = .07) or following recurrence (re-resection: 0.9 ± 0.7; median 1 [IQR 0-1] vs no re-resection: 0.5 ± 0.7; median 0 [IQR 0-1]; *P* = .10).

At last follow-up, deaths were more frequent in the no re-resection group (82.4%) than the re-resection group (63.0%); however, this difference was not statistically significant (*P* = .20).

### Re-Resection

Of those patients undergoing re-resection, 70.4% (19/27) had a GTR, 11.1% (3/27) had a NTR, and 18.5% (5/27) had a STR. 11.1% (3/27) underwent biopsy prior to a definitive re-resection. 74.1% (20/27) had only one definitive re-resection while 22.2% (6/27) went on to a second re-resection and 3.7% (1/27) went on to a fourth or fifth re-resection. Of note, one patient in the no re-resection group underwent two biopsies without proceeding to definitive resection.

### Survival

**Figure 1.**
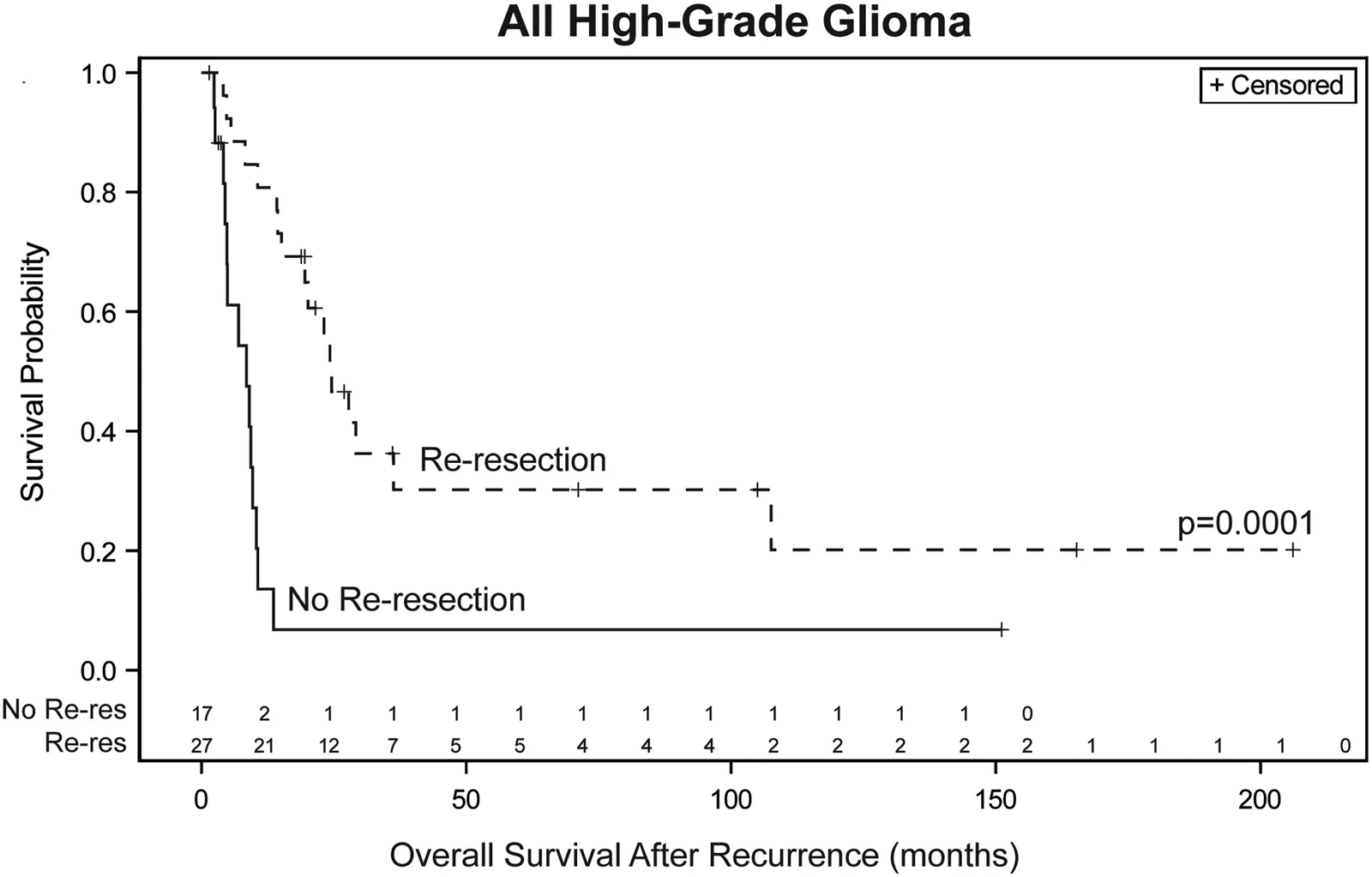
Overall survival from time of first recurrence to death by re-resection group.

**Figure 2.**
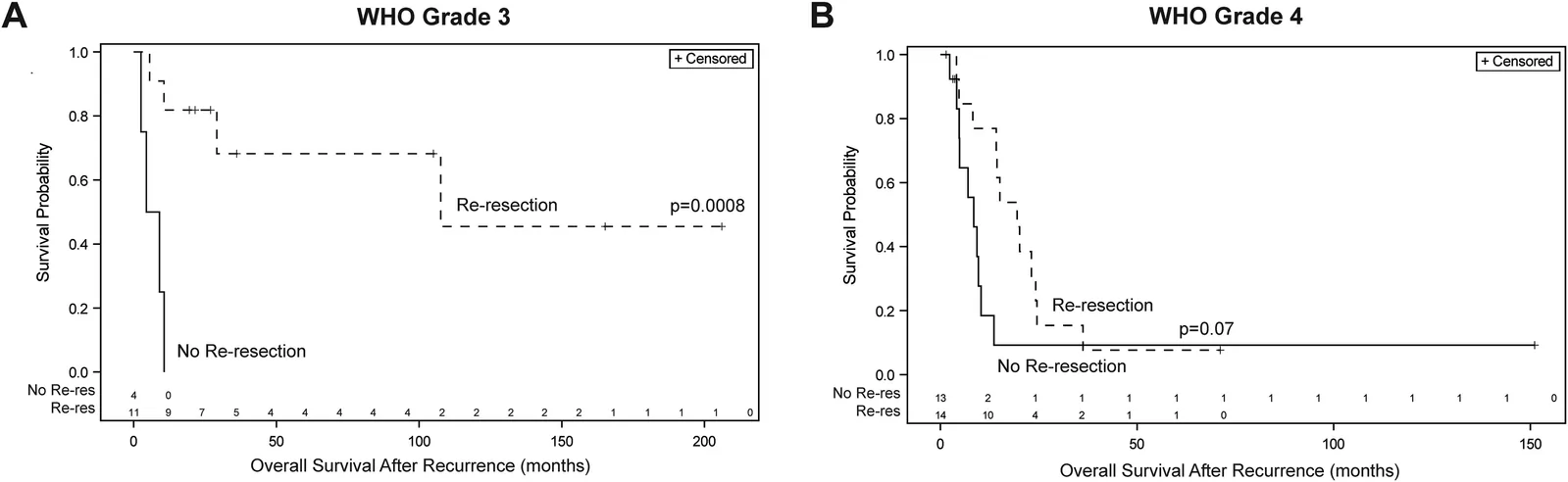
Overall survival from time of first recurrence to death by re-resection group for subgroup (A) WHO Grade 3 and (B) WHO Grade 4.

Kaplan-Meier analysis demonstrated that after first recurrence, the no re-resection group experienced limited long-term survival, whereas the re-resection group experienced a survival advantage (Figure 1; log-rank *P* = .0001). Median survival was 8.5 months with no re-resection (95% CI, 4.4-10.3) versus 24.6 months with re-resection (95% CI, 15.1-107.6). This difference was most pronounced in patients with Grade 3 tumors (Figure 2A; log-rank *P* = .0008). Median survival was 6.7 months with no re-resection (95% CI, 2.6-10.6) versus 107.6 months with re-resection (95% CI, 10.6-not estimable). In patients with Grade 4 tumors, re-resection did not reach statistical significance (Figure 2B; *P* = .07). Median survival was 8.5 months with no re-resection (95% CI, 4.1-10.3) versus 19.5 months with re-resection (95% CI, 8.2-24.2). Further analysis incorporating re-resection and tumor grade in a Cox proportional hazards model revealed a significant interaction (*P *= .036), suggesting tumor grade had an impact on response to re-resection. Among patients with Grade 3 tumors, re-resection was associated with a significantly lower hazard of death (HR 0.07, 95% CI, 0.02-0.32), whereas among patients with Grade 4 tumors, there was a similar direction of effect but without statistical significance (HR 0.50, 95% CI, 0.17-1.48).

Further analysis was performed to determine the impact of adjunct therapies on the response to re-resection. First, patients were stratified by BRAF status with “Positive” indicating treatment with a BRAF/MEK inhibitor and “Negative” indicating no treatment. The survival benefit of re-resection was maintained in both groups (Supplementary Figure S1, Negative *P *= .0006, Positive *P *= .03). Second, patients were stratified by radiation treatment after recurrence: re-resection only, radiation only, combination (re-resection + radiation), or neither. Kaplan-Meier analysis (Supplementary Figure S2) and Cox proportional hazard regressions identified significant differences between the treatment groups. Using the “Neither” group as a reference, the patients who received radiation only had no significant difference in survival (HR 1.42, 95% CI, 0.47-4.29; *P *= .537) whereas those who underwent re-resection only had an 80% lower hazard of death (HR 0.20, 95% CI, 0.06-0.67; *P *= .010). Patients treated with the combination of re-resection and radiation had a 68% lower hazard of death (HR 0.32, 95% CI, 0.13-0.80; *P *= .014) compared with those who received neither treatment. This demonstrates an association between re-resection and prolonged survival, regardless of concurrent radiation.

### Multivariate Models

In multivariable Cox regression adjusting for age at recurrence, sex, tumor grade, post-recurrence chemotherapy and radiotherapy regimen counts, and KL score at first recurrence, re-resection remained independently associated with improved survival, corresponding to a 73% lower hazard of death after recurrence (Table 2, HR 0.27, 95% CI, 0.10-0.69; *P* = .006). Older age at recurrence (>13 years) was associated with a higher hazard of death (HR 2.57, 95% CI, 1.07-6.20; *P* = .036). Sex, tumor grade, post-recurrence chemotherapy and radiotherapy exposure, and KL score at first recurrence were not significantly associated with survival in the adjusted model (all *P* > .05). Similar results were also obtained when adjusting for total chemotherapy and radiotherapy exposure (HR 0.26, 95% CI, 0.10-0.66; *P* = .005) and when adding BRAF status to the model (Supplementary Table S4, HR 0.25, 95% CI, 0.09-0.68; *P* = .006).

**Table 2.** Multivariable Cox proportional hazards model with re-resection, age at recurrence, sex, tumor grade, chemotherapy and radiotherapy regimens post-recurrence, and KL score at first recurrence

Covariate	HR (95% CI)	*P*-value
Re-resection (Yes vs No)	0.27 (0.10-0.69)	.006
Age at recurrence (>13 vs ≤13)	2.57 (1.07-6.20)	.036
Sex (Male vs Female)	0.85 (0.37-1.98)	.711
Tumor grade (3 vs 4)	1.63 (0.69-4.20)	.310
Number of chemotherapy regimens following recurrence	1.11 (0.88-1.40)	.367
Number of radiotherapy regimens following recurrence (0 vs ≥1)	1.02 (0.39-2.63)	.975
KL score at first recurrence	0.97 (0.93-1.01)	.121

Abbreviations: KL = Karnofsky/Lansky performance score.

### Propensity Score Matching

**Figure 3.**
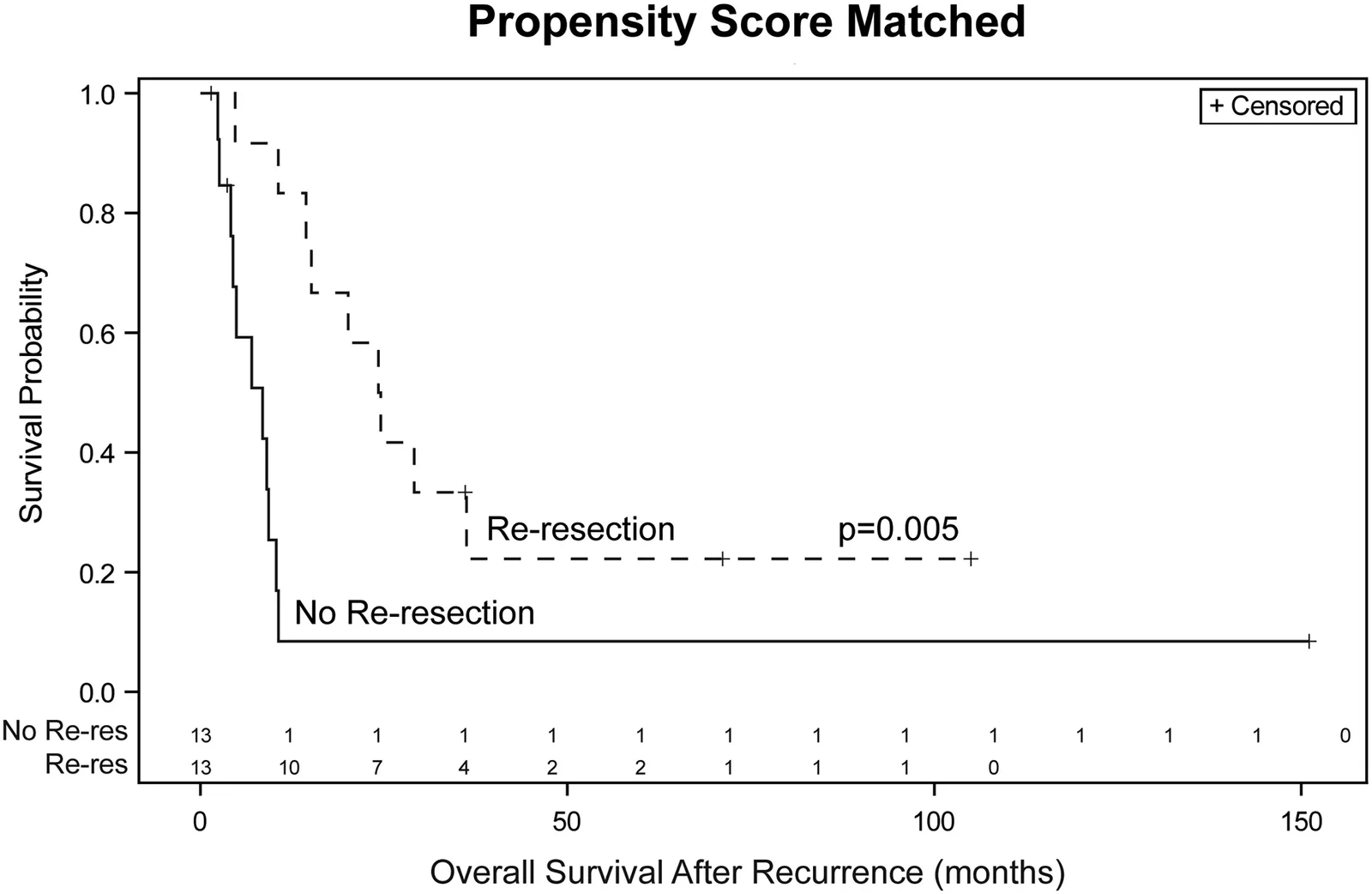
Overall survival from time of first recurrence to death by re-resection group for the propensity score matched cohort.

Propensity score matching (PSM) substantially improved covariate balance between the re-resection and no re-resection groups. The baseline covariates, including age category, sex, tumor grade, post-recurrence chemotherapy and radiation exposure, and KL score at first recurrence, achieved acceptable balance after matching (standardized mean differences ≤0.2). In the PSM cohort, re-resection remained significantly associated with improved survival after recurrence (Figure 3; HR 0.27, 95% CI, 0.11-0.68; *P* = .005). This suggests that, even after balancing baseline characteristics through matching, re-resection is linked to substantially improved post-recurrence survival.

## Discussion

OS in young patients with HGG is poor,^2^^,^^6^ and there are limited treatment options available at time of recurrence.^3^ A recent European survey of neuro-oncologists found that 92.4% of respondents would consider re-resection for a hemispheric HGG,^12^ but it is critically important that data guide clinical decision-making on further interventions. In designing the current study, we applied strict inclusion/exclusion criteria to allow us to address a directly translatable clinical question: Does re-resection of supratentorial HGG at first recurrence in young patients impact survival?

We chose to include only HGGs in young patients as previous studies have already demonstrated the value of re-resection at recurrence in other pediatric high-grade malignancies such as ependymoma^13^ and in adult HGGs.^8^ We identified a significant survival benefit to re-resection at recurrence in HGG. Further subset by WHO Grade, the survival benefit was most pronounced for Grade 3 tumors. There was not, however, a statistically significant difference for Grade 4 tumors. As such, this study is not able to fully address whether re-resection in Grade 4 tumors is associated with prolonged survival. While a larger sample size may aid in reaching significance in the future, the existing data still suggests any associated survival benefit for Grade 4 tumors will likely be lower than that noted for Grade 3 tumors. Prior studies in adult HGG also identify histologic and molecular characteristics which correlate with survival after re-resection in recurrence.^14^ We did note an enrichment in BRAF mutations and patients receiving BRAF/MEK inhibitors in the re-resection group; however, the survival benefit from re-resection was maintained when stratified by BRAF status and when BRAF status was included in the multivariate model.

Our study was also refined to supratentorial lobar tumors. By doing so, we specifically selected cases which are most amenable to GTR. We further selected only those with near total and GTR at initial resection. Of note, approximately 15% of each group required a “second-look” surgery to achieve initial NTR/GTR, but this was independent of recurrence/re-resection. A prior study^2^ did not find benefit to such “second-look” surgeries to obtain GTR in supratentorial tumors, though it did highlight a survival benefit when GTR was obtained during the initial resection. Applying our strict selection criteria provided an equivalent baseline for comparison; however, the results may not be directly applicable to HGGs in other locations or those for which only subtotal resection was performed at initial resection.

Previous studies in adult LGG and HGG have also demonstrated a correlation between extent of resection at re-resection with OS,^14^^,^^15^ but these studies may be confounded in part by differences in tumor location and surgical accessibility. We have addressed this with our strict selection criteria, but as a result, the sample size did not allow for further subset analysis based on extent of resection at re-resection.

Another critical consideration is complications and the long-term functional status of patients after re-resection. Previous studies in pediatric LGG^16^ and adult HGG^17^ report a similar low rate of complications with re-resection as with the initial resection and no decline in KPS after re-resection.^18^

Inherent to retrospective studies, there may also be a selection bias for those undergoing re-resection at time of recurrence. Our data did not distinguish the site of first recurrence from the site of the initial tumor; therefore, we cannot fully exclude bias derived from the recurrence occurring at a site less amenable to resection. We were, however, able to capture Karnofsky/Lansky performance status, and we found no difference in performance status at diagnosis or first recurrence between groups. To further address potential selection bias and confounding, we performed propensity score–matched analyses, which yielded consistent findings and demonstrated a persistent association between re-resection and improved survival after recurrence.

This work is limited by the relatively low number of patients, retrospective nature, and variability of adjuvant therapy regimens. The most definitive means to address the importance of re-resection of HGG on survival in young patients would be a randomized controlled trial; however, considering only approximately 200 hemispheric HGGs are diagnosed in young patients in the US per year,^1^ such a trial would face considerable challenges. This work provides strong evidence of survival benefit with re-resection in young patients with HGG, particularly WHO Grade 3 tumors. While there may be an associated benefit for Grade 4 tumors as well, this must be interpreted with caution for now due to the limitations of this study.

## Conclusion

This multi-center retrospective cohort study demonstrates that re-resection at first recurrence is associated with prolonged survival in supratentorial HGG in young patients. The effect is most pronounced in WHO Grade 3 gliomas and persists with multivariate analysis and propensity score matching. These findings support consideration of re-resection at time of first recurrence for surgically accessible HGG in young patients.

## Supplementary Material

vdag237_Supplementary_Data

## Data Availability

Anonymized data can be requested from the corresponding author.
