# Supplementary material for: Re-resection at first recurrence is associated with prolonged survival in young patients with high-grade glioma: A multi-center study
Source: Neurooncol Adv. 2026 Sep 7;8(1):vdag237. doi: 10.1093/noajnl/vdag237 (PMC13599782; doi:10.1093/noajnl/vdag237)
Supplement: Supplementary file 1 [file vdag237_supplementary_data.zip › Supplemental Table 4.docx]

**Supplemental Table 4:**

**Multivariable Cox proportional hazards model with re-resection, age at recurrence, sex, tumor grade, chemotherapy and radiotherapy regimens post-recurrence, KL score at first recurrence, and BRAF status**

| **Covariate (Including BRAF status)** | **HR (95% CI)** | **p-value** |
| --- | --- | --- |
| Re-resection (Yes vs No) | 0.25 (0.09–0.68) | 0.006 |
| Age at recurrence (>13 vs ≤13) | 2.38 (0.98–5.80) | 0.056 |
| Sex (Male vs Female) | 0.83 (0.36–1.91) | 0.654 |
| Tumor grade (3 vs 4) | 1.63 (0.61–4.38) | 0.329 |
| Number of chemotherapy regimens following recurrence | 1.02 (0.80–1.28) | 0.896 |
| Number of radiotherapy regimens following recurrence (0 vs ≥1) | 0.83 (0.31–2.19) | 0.698 |
| KL score at first recurrence | 0.95 (0.91–1.00) | 0.033 |
| BRAF status (Positive vs Negative) | 3.13 (1.04–9.43) | 0.043 |

Abbreviations: KL = Karnofsky/Lansky performance score
