# Supplementary material for: Re-resection at first recurrence is associated with prolonged survival in young patients with high-grade glioma: A multi-center study
Source: Neurooncol Adv. 2026 Sep 7;8(1):vdag237. doi: 10.1093/noajnl/vdag237 (PMC13599782; doi:10.1093/noajnl/vdag237)
Supplement: Supplementary file 1 [file vdag237_supplementary_data.zip › Supplemental Legends.docx]

**Supplemental Legends**

*Supplemental Figure 1*. Overall survival from time of first recurrence to death by re-resection group for subgroup (A) BRAF Negative and (B) BRAF Positive

[Alt Text: Kaplan-Meier survival curve comparing re-resection and no re-resection for BRAF negative and positive subgroups]

*Supplemental Figure 2*. Overall survival from time of first recurrence to death subset by re-resection and radiation status

[Alt Text: Kaplan-Meier survival curve comparing radiation only, re-resection only, combination, and neither]

*Supplemental Table 1*. Compiled clinical and molecular data
