# Supplementary material for: Re-resection at first recurrence is associated with prolonged survival in young patients with high-grade glioma: A multi-center study
Source: Neurooncol Adv. 2026 Sep 7;8(1):vdag237. doi: 10.1093/noajnl/vdag237 (PMC13599782; doi:10.1093/noajnl/vdag237)
Supplement: Supplementary file 1 [file vdag237_supplementary_data.zip › Supplemental Table 2.docx]

**Supplemental Table 2:
Re-resection group by institution**

| **Institution** | **Re-res (n=27)** | **No Re-res (n=17)** |
| --- | --- | --- |
| Children’s of Alabama | 3 (11.1%) | 4 (23.5%) |
| Children’s Hospital Colorado | 3 (11.1%) | 5 (29.4%) |
| Ann & Robert H. Lurie Children’s Hospital of Chicago | 3 (11.1%) | 2 (11.8%) |
| Phoenix Children’s Hospital | 1 (3.7%) | 1 (5.8%) |
| UCLA Mattel Children’s Hospital | 1 (3.7%) | 0 (0.0%) |
| Monroe Carell Jr. Children’s Hospital at Vanderbilt | 7 (25.9%) | 1 (11.8%) |
| St. Louis Children’s Hospital | 9 (33.3%) | 3 (16.7%) |

Categorical p-value from Fisher’s exact tests (p=0.57).
