# Supplementary material for: Re-resection at first recurrence is associated with prolonged survival in young patients with high-grade glioma: A multi-center study
Source: Neurooncol Adv. 2026 Sep 7;8(1):vdag237. doi: 10.1093/noajnl/vdag237 (PMC13599782; doi:10.1093/noajnl/vdag237)
Supplement: Supplementary file 1 [file vdag237_supplementary_data.zip › Supplemental Table 3.docx]

**Supplemental Table 3:
Molecular information by re-resection group**

|  |  | **Mutated** | **WT** | **Not Specified** |
| --- | --- | --- | --- | --- |
| **H3K27M** | **Re-res** | 2^a^ (7.4%) | 1 (3.7%) | 24 (88.9%) |
|  | **No Re-res** |  | 1 (5.9%) | 16 (94.1%) |
|  |  |  |  |  |
|  |  |  | ^a^Includes 1 "K27me3 loss" \| ^a^Includes 1 "H3K27me3 loss" |  |
|  |  | **Mutated** | **WT** | **Not Specified** |
| **H3 G34** | **Re-res** | 2 (7.4%) |  | 25 (92.6%) |
|  | **No Re-res** | 2 (11.8%) | 1 (5.9%) | 14 (82.4%) |
|  |  |  |  |  |
|  |  | **Mutated** | **WT** | **Not Specified** |
| **BRAF** | **Re-res** | 5^b^ (18.5%) | 3 (11.1%) | 19 (70.4%) |
|  | **No Re-res** | 1 (5.9%) | 3 (17.6%) | 13 (76.5%) |
|  |  | ^b^Includes 1 EMLA-BRAF Fusion | |  |
|  |  |  |  |  |
|  |  | **Mutated** | **WT** | **Not Specified** |
| **ATRX** | **Re-res** | 2 (7.4%) | 1 (3.7%) | 24 (88.9%) |
|  | **No Re-res** | 3^c^ (17.6%) | 1 (5.9%) | 13 (76.5%) |
|  |  | ^c^Includes 1 "loss" |  |  |
|  |  |  |  |  |
|  |  | **Mutated** | **WT** | **Not Specified** |
| **IDH1** | **Re-res** | 1 (3.7%) | 9 (33.3%) | 17 (63.0%) |
|  | **No Re-res** | 1 (5.9%) | 4 (23.5%) | 12 (70.6%) |
|  |  |  |  |  |
|  |  | **Methylated** | **Unmethylated** | **Not Specified** |
| **MGMT** | **Re-res** | 1 (3.7%) | 5 (18.5%) | 21 (77.8%) |
|  | **No Re-res** |  | 2 (11.8%) | 15 (88.2%) |
|  |  |  |  |  |
|  |  | **Loss** |  | **Not Specified** |
| **CDKN2A** | **Re-res** | 4 (14.8%) |  | 23 (85.2%) |
|  | **No Re-res** | 2 (11.8%) |  | 15 (88.2%) |
|  |  |  |  |  |
|  |  | **Mutated** |  | **Not Specified** |
| **p53** | **Re-res** | 9^d^ (33.3%) |  | 18 (66.7%) |
|  | **No Re-res** | 6^e^ (35.3%) |  | 11 (64.7%) |
|  |  | ^d^Includes 2 "loss" and 2 "partial" | |  |
|  |  | ^e^Includes 1 "17p-deleted" and 1 "partial" | |  |
